# Impact of Pre- and Post-Dilatation on Long-Term Outcomes After Self-Expanding and Balloon-Expandable TAVI

**DOI:** 10.3390/jfb16080282

**Published:** 2025-08-01

**Authors:** Alexandru Stan, Ayman Elkahlout, Marius Mihai Harpa, Marian Pop, Mihaly Veres, Antonela Delia Stan, Paul-Adrian Călburean, Anda-Cristina Scurtu, Klara Brînzaniuc, Horatiu Suciu

**Affiliations:** 1 Doctoral School, George Emil Palade University of Medicine, Pharmacy, Science and Technology of Târgu Mureş, 540142 Târgu Mureş, Romania; 2 Department of Interventional Cardiology, Emergency Institute for Cardiovascular Diseases and Transplantation, 540136 Târgu Mureș, Romania; 3 Department of Surgery IV, George Emil Palade University of Medicine, Pharmacy, Science and Technology of Târgu Mureș, 540142 Târgu Mureș, Romania; 4 Department of Cardiovascular Surgery, Emergency Institute for Cardiovascular Diseases and Transplantation, 540136 Târgu Mureș, Romania; 5 Department of Radiology, George Emil Palade University of Medicine, Pharmacy, Science and Technology of Târgu Mureș, 540142 Târgu Mureș, Romania; 6 Department of Intensive Care, Emergency Institute for Cardiovascular Diseases and Transplantation, 540136 Târgu Mureș, Romania; 7 Faculty of Law, University of Bucharest, 030018 Bucharest, Romania; 8 Department of Biostatistics and Medical Informatics, George Emil Palade University of Medicine, Pharmacy, Science and Technology of Târgu Mureş, 540142 Târgu Mureş, Romania; 9 Department of Anatomy, George Emil Palade University of Medicine, Pharmacy, Science and Technology of Târgu Mureș, 540142 Târgu Mureș, Romania

**Keywords:** severe aortic stenosis, transcatheter aortic valve implantation, balloon-expandable valve, self-expandable valve

## Abstract

The main objective of this study was to compare the long-term outcomes of transcatheter aortic valve implantation (TAVI) in patients with severe aortic stenosis, focusing on differences between self-expanding valve (SEV) versus balloon-expandable valve (BEV) prostheses and the influence of balloon pre- and post-dilatation on clinical results. The secondary objective was to report the long-term outcomes after TAVI in Romania. All patients who underwent a TAVI procedure for severe AS between November 2016 and May 2025 at a tertiary center in Romania were included in the present study. A total of 702 patients were included, of which 455 (64.8%) and 247 (35.1%) patients received a BEV (Sapien3 platform) and a SEV (Accurate, Boston, Portico, Evolut, or Navitor platforms), respectively. Pre-dilatation was performed in 514 (73.2%) cases, and post-dilatation was performed in 189 (26.9%) cases. There were 10.5 and 7.8 all-cause and cardiovascular-cause mortality event rates per 100 patient years, respectively. In regard to the univariable Cox regression, a BEV has significantly lower mortality than an SEV (HR = 0.67[0.46–0.96], *p* = 0.03), pre-dilatation did not influence mortality (HR = 0.71[0.48–1.04], *p* = 0.08), and post-dilatation significantly increased mortality (HR = 1.51[1.05–2.19], *p* = 0.03). In regard to the multivariable Cox regression, survival was not influenced by pre-dilatation or the valve platform, while post-dilatation had a trend towards higher mortality (*p* = 0.06). The BEV and SEV have similar survival rates, with no heterogeneity among a large number of TAVI platforms. While pre-dilatation had no impact on mortality, post-dilatation was associated with a trend towards increased mortality (*p* = 0.06), which was independent of the transprosthetic gradient. Survival after TAVI in Romania is comparable to that reported in Western registries.

## 1. Introduction

Severe aortic stenosis (AS) is a common valvular disease in the elderly and is associated with a poor prognosis once symptoms develop, with approximately 50% mortality at 2 years in the absence of aortic valve replacement [1,2]. Transcatheter aortic valve implantation (TAVI) has revolutionized the management of severe AS over the past two decades, providing a less invasive alternative to surgical aortic valve replacement (SAVR) for symptomatic patients across a wide spectrum of surgical risks [3]. Multiple randomized trials in high-, intermediate-, and low-risk populations have established TAVI as an effective therapy that improves survival and quality of life, leading to its incorporation as a first-line treatment for many patients with severe AS into the current guidelines [4].

Two main categories of transcatheter heart valves are in clinical use: balloon-expandable valves (BEVs) and self-expanding valves (SEVs). BEVs rely on balloon inflation to deploy the valve, whereas SEVs (typically mounted on a nitinol frame) auto-expand within the annulus [4]. Both valve types have undergone iterative improvements, and studies have found no significant differences in early mortality between BEV and SEV TAVI platforms [4]. However, BEVs tend to be associated with lower rates of paravalvular regurgitation, stroke, and new conduction disturbances, requiring permanent pacemakers, while SEVs often achieve larger post-procedural valve orifice areas, with lower transvalvular gradients [5]. These differences underscore the importance of valve selection on a case-by-case basis.

Another procedural consideration in regard to TAVI is the use of adjunct balloon dilations. Pre-dilatation was historically routine to facilitate valve crossing and expansion [3,6]. On the other hand, balloon post-dilatation of the transcatheter valve after deployment is sometimes performed to improve valve expansion and reduce paravalvular leaks, but this step is applied selectively, due to safety considerations [7]. Notably, patients requiring post-dilation have been observed to experience higher rates of periprocedural stroke, reflecting the potential risk of additional balloon inflations [8]. Accordingly, current practice adopts an individualized approach, while pre- and post-dilatation are used selectively based on the patient’s anatomy and intraprocedural results [3]. The ongoing debate regarding the optimal use of balloon dilatation strategies highlights the need for clarity on their long-term impact.

In this context, we aimed to compare the long-term outcomes of TAVI in patients with severe AS, focusing on differences between self-expanding versus balloon-expandable valve prostheses and the influence of balloon pre- and post-dilatation on clinical results. Also, we aimed to report the long-term outcomes after TAVI in Romania, as such survival reports from Eastern Europe are currently under-represented. Moreover, reports on morbidity and mortality in cardiovascular diseases from Eastern Europe usually rely on aggregated country-specific data and not individual patient-level data [9,10].

## 2. Materials and Methods

The research protocol complied with the Declaration of Helsinki and was approved by the local Ethics Committees at the Emergency Institute for Cardiovascular Diseases and Transplantation Târgu Mureș (approval number 2263/22 April 2025).

All patients who underwent the TAVI procedure for severe degenerative aortic stenosis at our tertiary center were selected for inclusion in the present study. Our institutional protocol involves the Heart Team (cardiologist [interventional and echography specialist], cardiac surgeon, and anesthetist) that decides between the use of SAVR or TAVI, based on surgical risk scores and other relevant criteria. The exclusion criteria consisted of (1) an age less than 18 years, (2) non-degenerative severe aortic stenosis (e.g., congenital), or (3) the recipient of a TAVI intervention for aortic regurgitation or a valve-in-valve TAVI procedure. The inclusion criteria consisted of (1) the recipient of a TAVI performed during November 2016–May 2025 and (2) available for follow-up as per the Romanian National Health Insurance System database. The Romanian National Health Insurance System database supplied the mortality status for all the patients. For patients who had died during follow-up, the Romanian National Institute of Statistics provided the exact date and cause of death, according to the tenth revision of the International Classification of Diseases (ICD-10). If the cause of death belonged to diseases of the circulatory system, then death was considered to be due to a cardiovascular cause. Data regarding anthropometrics, relevant medical history, clinical status at hospital admission, chronic medical treatment, routine laboratory parameters, and echocardiographic parameters were collected. Electronic health records provide detailed hospitalization costs. Data regarding detailed interventional parameters were also collected. The pre- and post-dilatation decision was at the discretion of the operator, on a case-by-case basis, based on the perceived valve expansion and stability, the occurrence of para- or intra-valvular leaks, and the intraoperative transprosthetic gradient. 

A significance threshold (α) of 0.05 and a 95% confidence interval (CI) were applied. The normality of continuous variables was assessed using the Shapiro–Wilk test. Variables with a normal (parametric) distribution were expressed as the mean ± standard deviation and compared using the unpaired Student’s *t*-test. Non-normally distributed (non-parametric) continuous variables and discrete variables were presented as the median (interquartile range) and compared using the Mann–Whitney U test. Categorical variables were summarized as counts and percentages, with comparisons made using Fisher’s exact test. Survival analysis was conducted using Kaplan–Meier curves, and differences between groups were evaluated using the log-rank test. To assess the influence of various predictors on survival, both univariable and multivariable Cox proportional hazards models were employed, reporting associations as hazard ratios (HRs). Multivariable models included a minimum of 15 events per covariate to ensure model stability [11]. The models were developed using a stepwise approach, and the risk of overfitting was evaluated by calculating Akaike’s Information Criterion (AIC) at each step [12]. The AIC was used to determine whether additional variables improved model performance or induced overfitting. Missing data were imputed using the population median method [13]. All the statistical analyses were performed using Python version 3.9.13 and matplotlib, lifelines, and SciPy libraries.

## 3. Results

**Figure 1:**
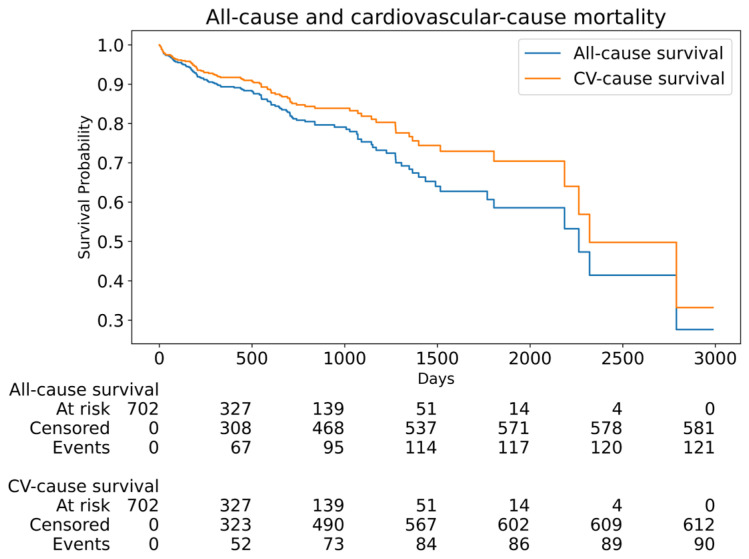
All-cause and cardiovascular-cause mortality in the studied population.

**Figure 2:**
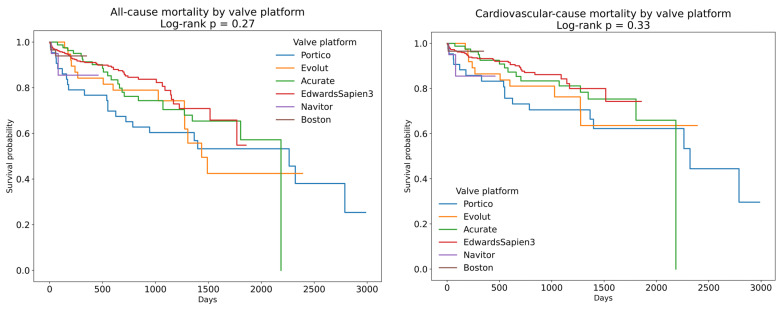
Survival curves for each TAVI platform.

**Figure 3:**
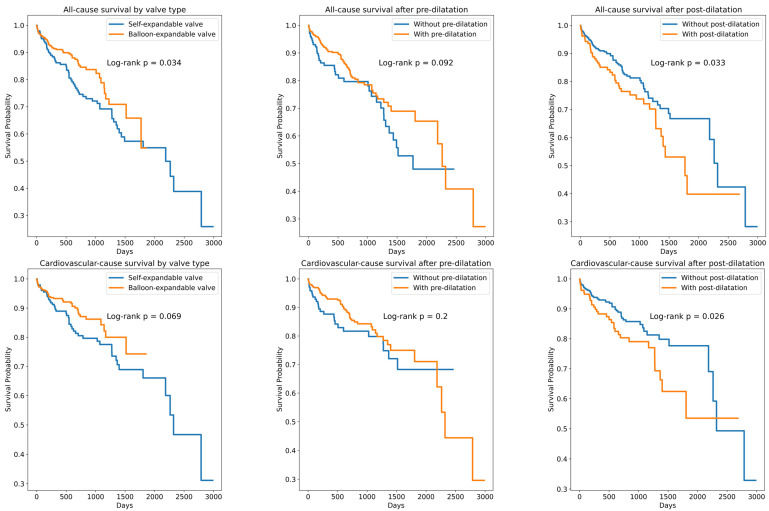
Kaplan–Meier survival curves comparison after valve implantation, pre-dilatation, and post-dilatation.

**Figure 4:**
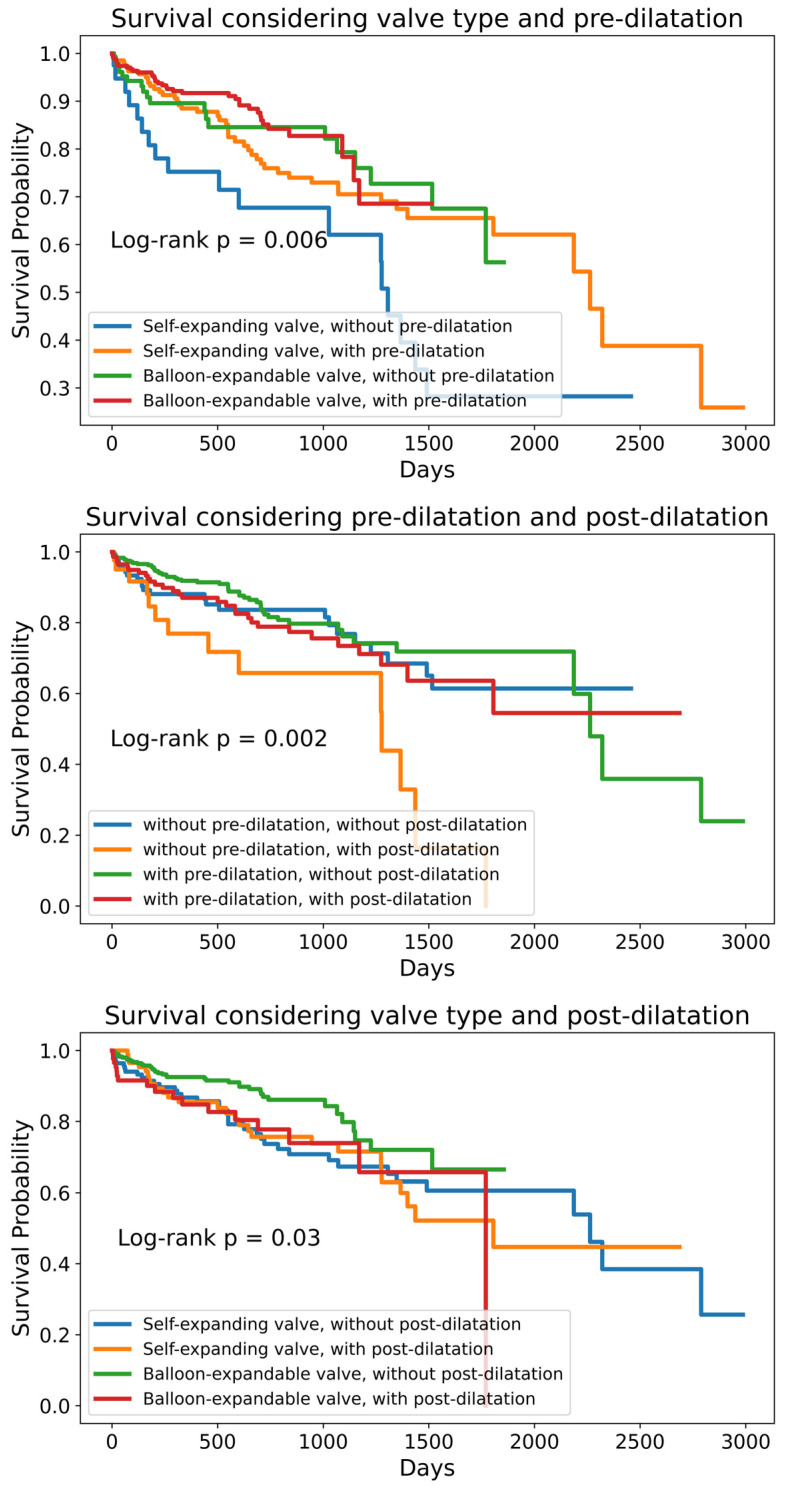
Kaplan–Meier survival curves comparison considering valve type, pre-dilatation, and post-dilatation.

A total of 702 patients who underwent TAVI for severe degenerative aortic stenosis were included in this study. The baseline clinical and echocardiographic characteristics are summarized in Table 1. The median age of the cohort was 79 (75–83) years, and 380 patients (54.1%) were male. Balloon-expandable valves (BEVs) were implanted in 455 patients (64.8%), while self-expanding valves (SEVs) were used in 247 patients (35.1%). Pre-dilatation was performed in regard to 514 patients (73.2%), and post-dilatation in regard to 189 patients (26.9%). There were no significant differences in the length of stay between valve types (median 6.90 [5.20–9.91] days for SEVs vs. 6.87 [4.98–9.70] days for BEVs, *p* = 0.24). In-hospital mortality was 1.6% (n = 11), and the rate of permanent pacemaker implantation was 8.1% (n = 57). Over a median follow-up of 1.42 (0.43–2.41) years, 92 (13.1%) and 123 (17.5%) patients died of cardiovascular and all-cause causes, respectively (Figure 1 and Figure 2). There were 10.5 all-cause mortality event rates per 100 patient years and 7.8 cardiovascular-cause mortality event rates per 100 patient years. The 1-year, 2-year, 3-year, 4-year, and 5-year all-cause survival rates were 87.4%, 80.1%, 69.5%, 54.7%, and 50.9%, respectively. The 1-year, 2-year, 3-year, 4-year, and 5-year cardiovascular-cause survival rates were 90.1%, 83.8%, 73.0%, 61.3%, and 55.4%, respectively. The Kaplan–Meier curves demonstrated higher survival among patients who received BEVs compared with SEVs (log-rank *p* = 0.02), as well as lower survival among those undergoing post-dilatation (log-rank *p* = 0.03); survival did not differ significantly according to the pre-dilatation status (log-rank *p* = 0.08) (Figure 3). Similarly, all subgroups with post-dilatation were associated with lower survival (Figure 4).

In regard to the univariable Cox regression, BEV implantation was associated with a significantly lower risk of death versus SEV. Pre-dilatation was not significantly associated with mortality, whereas post-dilatation was associated with increased all-cause mortality (Table 2). Other valve platforms (Accurate, Boston, Portico, Evolut, Navitor) did not reach statistical significance for all-cause mortality reduction, although Portico tended toward higher risk (*p* = 0.07). A total of 57 patients (8.1%) required a new permanent pacemaker implantation. In regard to the univariable logistic regression, neither pre-dilatation nor post-dilatation was significantly associated with pacemaker implantation (Table 2). However, the use of the Portico or the Navitor platforms was a significant predictor of pacemaker implantation. In contrast, the Accurate and Boston platforms were not associated with higher pacemaker risk (Table 2). Compared with patients receiving SEVs, those who received BEVs were slightly younger and more frequently male. The left ventricular ejection fraction (LVEF) was lower in the BEV group, and the aortic valve area was larger (0.75 cm^2^ ± 0.16 vs. 0.60 [0.54–0.70] cm^2^, *p* = 0.009). The annular dimensions tended to be greater in BEV patients: median annulus area 477.2 mm^2^ (420.2–546.5) versus 417.0 mm^2^ (365.0–463.0) for SEV (*p* < 0.0001), and annulus perimeter 79.1 (74.0–84.3) mm versus 73.8 ± 6.8 mm (*p* < 0.0001). The BEV group also had larger sinus of Valsalva diameters (30.9 mm ± 3.3 vs. 27.7 [26.2–30.2] mm, *p* < 0.0001) and higher sinotubular junction diameters (28.9 mm ± 3.3 vs. 26.6 [24.8–30.0] mm, *p* = 0.0016). 

**Figure 5:**
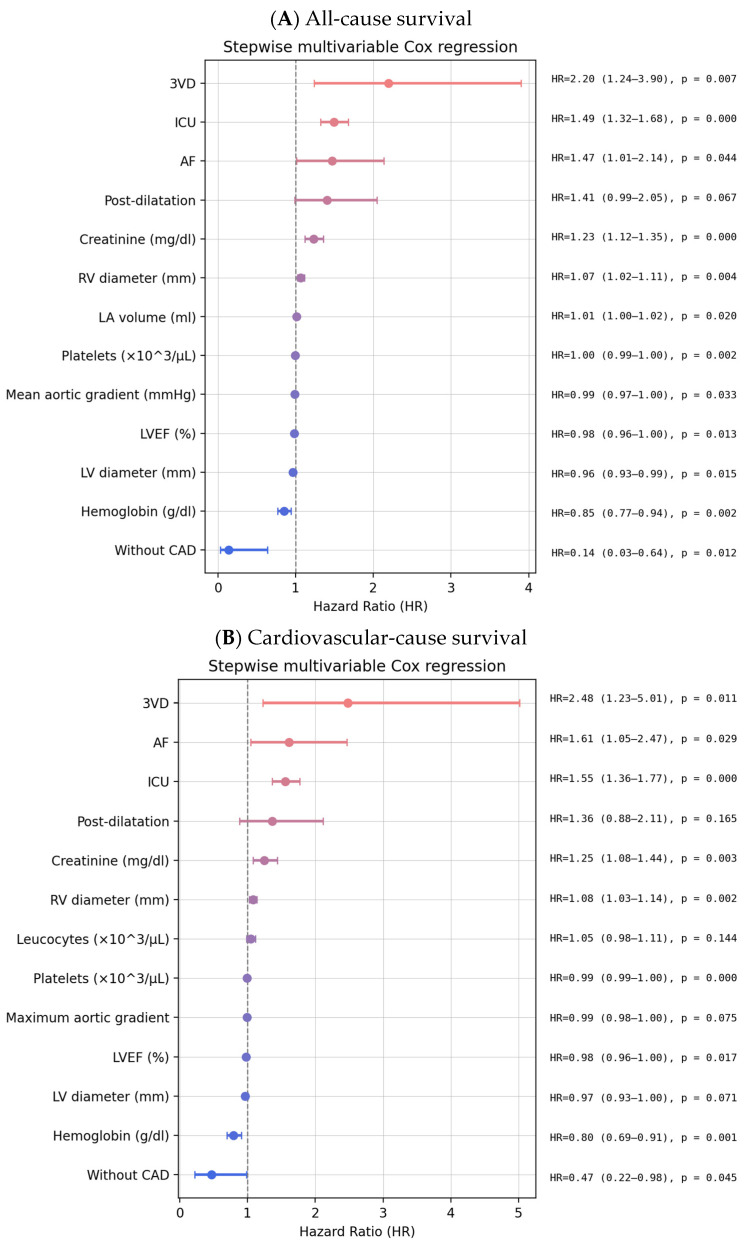
Multivariable analysis of long-term survival. Notes: 3VD—three-vessel disease; AF—atrial fibrillation; CAD—coronary artery disease; ICU—intensive care unit stay; LA—left atrium; LBBB—left bundle branch block; LV—left ventricle; LVEF—LV ejection fraction; RV—right ventricle.

Logistic regression was used to identify independent predictors of BEV selection: a lower LVEF, a smaller annulus area, and a lower LCA height (Table 3). Stepwise multivariable logistic regression identified dilated cardiomyopathy as a strong negative predictor of pre-dilatation and higher maximum aortic velocity as a positive predictor. For post-dilatation, a history of coronary artery bypass grafting, a higher mean gradient, and the presence of aortic regurgitation increased the likelihood of post-dilatation, whereas a larger sinotubular junction height reduced it (Table 3). The presence of three-vessel coronary artery disease, atrial fibrillation, ICU stay, and higher creatinine is associated with all-cause mortality, while a higher LVEF, a higher LV diameter, and the absence of coronary artery disease had a protective effect against all-cause mortality (Figure 5). Post-dilatation had a trend towards increasing all-cause mortality, but it did not reach statistical significance (*p* = 0.06). Interestingly, the use of a BEV or SEV did not impact mortality. There was no significant difference in pre- or post-dilatation rates among the operators, nor in the valve choice among operators.

## 4. Discussion

The main findings of this study can be summarized as follows: (1) This is the first long-term survival analysis from Romania, which reports rates of all-cause and cardiovascular-cause mortality that are comparable with other Western real-world registries and randomized controlled trials; (2) in regard to the univariable analysis, BEVs are associated with higher survival than SEVs and the use post-dilatation is associated with lower survival, while the use of pre-dilatation did not influence survival; (3) however, in regard to the multivariable analysis, survival was impaired by complex CAD, atrial fibrillation, ICU stay, and higher creatinine, while post-dilatation showed a trend towards increased mortality (*p* = 0.06), independent of the transprosthetic gradient; and (4) this study included a large number of TAVI platforms (six platforms in total) and there was no heterogeneity in the survival outcomes, while the Portico and Navitor valves were associated with a higher likelihood of permanent pacemaker implantation.

Large-scale studies and meta-analyses indicate that long-term survival after TAVI is comparable between BEVs and SEVs. In pooled randomized trials and propensity-matched studies of newer-generation devices, there were no significant differences in the 30-day or 1-year mortality between BEV and SEV implants [4]. This aligns with our findings and reinforces the finding that valve choice (BEV vs. SEV) does not substantially impact early survival outcomes. Also, randomized trials with a longer follow-up confirmed that BEV and SEV implants have similar outcomes [14,15]. In the clinical context of systolic dysfunction, the BEV was reported to have higher 5-year survival rates than the SEV [16]. However, important differences emerge in regard to secondary endpoints. SEV TAVI tends to achieve larger effective orifice areas and lower transvalvular gradients than BEV TAVI, which can be advantageous in patients with small annuli [4]. The trade-off is a higher incidence of paravalvular regurgitation with SEVs, particularly older-generation designs, as demonstrated in meta-analyses. BEVs, with their inflatable skirts, are associated with significantly lower rates of moderate-to-severe paravalvular leakage [4,17]. The association of SEVs with larger effective orifice areas is also observed in our study. While valve choice is at the discretion of the operator, patients with a small annulus received an SEV more often than a BEV (Table 1). In contrast, patients with a BEV more often had a lower LVEF, and a higher incidence of dilated cardiomyopathy. However, the final transprosthetic gradient did not differ among the valve platforms.

Another key difference is the rate of conduction disturbances requiring permanent pacemakers. Consistently, BEVs have shown lower pacemaker implantation rates compared to SEVs [17]. For example, a recent meta-analysis of new-generation valves reported pacemaker rates of around 11.5% with the Sapien3 platform (BEV) versus 16.9% with the Medtronic Evolut platform (SEV) [4]. The ACURATE neo (SEV) was a notable exception, with pacemaker needs (9–10%) even lower than Sapien3 in some studies. Nonetheless, the overall trend across both trials and real-world registries is that self-expanding valves, especially those with deeper supra-annular frames like the CoreValve/Evolut platform, carry a higher risk of atrioventricular blockage and permanent pacemaker implantation. Stroke rates, by contrast, have been found to be broadly similar between BEV and SEV platforms in most contemporary analyses. Overall, our comparative outcomes for BEV versus SEV TAVI are in line with the literature: survival is equivalent, but device selection influences hemodynamic and complication profiles, with BEVs favoring fewer leaks and conduction issues, and SEVs offering larger valve areas, at the possible cost of more pacemakers being required.

We evaluated the impact of pre-dilatation (balloon aortic valvuloplasty before TAVI) and found that routine pre-dilatation may not be necessary in all cases. This is supported by current evidence: randomized trials (including those with Sapien3 and Evolut valves) and meta-analyses have demonstrated that omitting pre-dilatation, the “direct TAVI” approach, is feasible and non-inferior to the standard strategy involving pre-ballooning [18]. Thus, skipping the valvuloplasty step did not compromise device success or early clinical outcomes in appropriately selected patients [6]. Our practice of selective pre-dilatation is consistent with these findings. Direct TAVI offers procedural advantages, such as a shorter procedure time and less contrast use, and it avoids the potential downsides of an extra balloon inflation (e.g., annular injury or debris embolization) [19]. Importantly, there is a suggestion from large registries that direct TAVI might even reduce the risk of conduction disturbances. The UK TAVI registry and a recent meta-analysis both observed a trend toward lower permanent pacemaker implantation rates when TAVI was performed without pre-dilatation [20]. Although this was not a randomized comparison, it is clinically plausible that avoiding the initial balloon inflation might lessen trauma to the conduction system. Thus, the current best practice is to individualize the use of pre-dilatation. In patients with a favorable anatomy (e.g., less calcified, non-bicuspid valves, good crossability), many operators now proceed with direct TAVI safely [18]. Conversely, heavy calcification, a very tight or bicuspid valve, or uncertainty about crossing may still warrant pre-dilatation to ensure the valve can be delivered and expanded properly. In our study, stepwise multivariable logistic regression identified dilated cardiomyopathy as a strong negative predictor of pre-dilatation and higher maximum aortic velocity as a positive predictor (Table 3). While the pre-dilatation decision was at the discretion of the operator on a case-by-case basis, these results suggest that cases with a higher maximum aortic velocity undergo pre-dilatation more often, possibly due to expected valve crossing difficulties, and cases involving dilated cardiomyopathy undergo pre-dilatation less often, due to a preference for an expedited procedure as such patients have a higher risk of procedural complications. Our results support a personalized approach: the absence of a consistent outcome impact due to pre-dilatation suggests that it can be omitted in many cases, as the simplified direct TAVI does not sacrifice safety.

Balloon post-dilatation is often performed to optimize the final result after valve deployment, but our analysis suggests it should be used cautiously. Post-dilatation can significantly reduce residual paravalvular regurgitation and improve prosthesis expansion, which is beneficial for valve function. However, the trade-offs are non-negligible. Mechanically dilating the valve post-implant can destabilize atherosclerotic debris or calcifications, potentially increasing stroke risk. Indeed, evidence from at least one center showed that requiring post-dilatation was an independent predictor of acute stroke after TAVI [21]. In that study, the 30-day stroke incidence was 3.7%, and post-dilatation emerged as a significant risk factor for periprocedural stroke [21]. This finding underscores a known safety concern: additional ballooning may dislodge embolic material, despite the use of embolic protection devices in some cases. We also considered whether post-dilatation affects survival; while there is no evidence post-dilating in itself alters long-term mortality, an associated stroke or complication can worsen outcomes. Furthermore, post-dilatation can impact conduction safety. The additional radial force on the annulus can exacerbate trauma to the AV node or His bundle. Some reports have linked balloon post-dilating with higher rates of new pacemaker implantation [18]. Our data did not show a significant difference in survival between patients who underwent post-dilatation and those who did not, but given the above risks, this step should be performed only for clear indications (e.g., unacceptable moderate/severe paravalvular leakage or a high residual gradient). Thus, post-dilatation remains a useful adjunct to improve valve sealing and gradients when needed, but the decision must balance its benefits with the small but real increase in procedural risk (notably, stroke and conduction blockage leading to permanent pacemaker implantation). In our study, in regard to the stepwise multivariable logistic regression, a history of coronary artery bypass grafting, a higher mean gradient, and the presence of aortic regurgitation increased the likelihood of post-dilatation, whereas a larger sinotubular junction height reduced it (Table 3). Similarly, the post-dilatation decision was at the discretion of the operator on a case-by-case basis, based on the intraoperative transprosthetic gradient and perceived valve stability after implantation. These results suggest that patients with higher mean aortic gradients and aortic regurgitation tend to have impaired valve stability or higher intraoperative gradients and require post-dilatation. Our practice of careful hemodynamic assessment and selective post-dilatation is aligned with current recommendations to minimize unnecessary balloon inflations [3].

The extended follow-up of our TAVI cohort enables a comparison to be conducted with international benchmarks. At 1 year, survival in our study approximated the mid-80% to 90% range (depending on the risk profile), which is consistent with contemporary registries and trials. For instance, in intermediate-risk patients as part of the PARTNER 2 trial, one-year survival was about 89% and, even in high-risk cohorts, a one-year survival of 80% has been reported (e.g., CoreValve US Pivotal trial), with our outcomes being within these ranges [22,23,24]. In high-risk elderly populations, long-term mortality is heavily influenced by comorbid conditions. The PARTNER-1 trial (high-risk operable patients) demonstrated a 68% mortality by 5 years in TAVI patients [25]. Similarly, the NOTION and CoreValve high-risk trials have shown 40–45% five-year survival for TAVI, with no significant difference versus surgery [26,27]. Our 5-year all-cause mortality falls in line with these figures, suggesting that our patient cohort did not experience any excess mortality compared to the international experience. Notably, as patient risk profiles improve (intermediate or low surgical risk), long-term survival after TAVI has correspondingly improved. The SURTAVI trial (intermediate-risk profile) reported about 69% of TAVI patients free from death or disabling stroke at 5 years, similar to SAVR outcomes [28]. That translates to approximately 70% 5-year survival in that moderate-risk group, highlighting that TAVI can confer durable benefits well beyond the first year for a broad range of patients. Our cohort’s 5-year survival rate sits between the high-risk and intermediate-risk trial benchmarks, as expected given our mix of risk profiles. This comparison to global data provides reassurance that there has been no late divergence in survival unique to our practice; in fact, it reinforces the durability of TAVI outcomes that are now documented across multiple populations, which is particularly important since populations from Eastern Europe are known to have high long-term mortality in regard to cardiovascular diseases [29,30]. We also note that the causes of late death in TAVI populations are often non-cardiac (e.g., cancer, lung disease), reflecting the advanced age of these patients; this provides context in regard to why even with contemporary valve therapy, the five-year mortality can approach 50% in real-world registries. Importantly, our findings of comparable long-term survival between BEV and SEV TAVI are also supported by trial data, indicating no device-specific divergence up to 5 years [25]. In conclusion, our TAVI survival outcomes are comparable to those reported in international trials and registries. However, the cost effectiveness of TAVI compared to SAVR in Romania differs from that observed in Western countries. Our previous analysis showed that TAVI is more cost effective for patients with high surgical risk, whereas SAVR remains more economically favorable for those with low-to-intermediate surgical risk. This contrasts with findings from other studies, which have suggested that TAVI is cost efficient, even in intermediate-risk patients [31,32]. 

The present study has several strengths and limitations. The main limitation of this study is its retrospective nature, and certain variables were missing (e.g., STS score). Also, our study is a non-randomized observational study, which inherently carries a risk of residual confounding. We employed multivariate adjustments to account for baseline differences, but unmeasured factors could have influenced the outcomes. However, the study included a relatively large number of patients, with accurate follow-up provided by the Romanian National Institute of Statistics. Also, this study includes a large number of valve platforms, especially for the SEV.

## 5. Conclusions

The present study is the first long-term survival analysis from Romania, reporting rates of all-cause and cardiovascular-cause mortality that are comparable with other Western real-world registries and randomized controlled trials. In regard to the univariable analysis, the BEV was associated with a higher level of survival than the SEV, and the use of post-dilatation was associated with lower survival, while the use of pre-dilatation did not influence survival. However, in regard to the multivariable analysis, survival was impaired by complex CAD, atrial fibrillation, ICU stay, and higher creatinine, while post-dilatation showed a trend towards increased mortality. 

## Figures and Tables

**Table 1:** Clinical characteristics of the studied population.

Parameter	All Patients (n = 702)	Self-Expanding Valve (n = 245)	Balloon-Expanding Valve (n = 457)	*p*-Value
Age (years)	79 (75–83)	80 (76–84)	78 (74–82)	0.0001
Male sex	380 (54.1%)	100 (40.5%)	280 (61.5%)	0.0001
Hospitalization (days)	6.88 (5.02–9.77)	6.90 (5.20–9.91)	6.87 (4.98–9.70)	0.24
Diabetes mellitus	256 (36.4%)	93 (37.5%)	163 (35.7%)	0.68
Hypertension	603 (85.7%)	223 (89.9%)	380 (83.3%)	0.01
Atrial fibrillation	250 (35.5%)	77 (31.0%)	173 (37.9%)	0.07
Chronic kidney disease	128 (18.2%)	44 (17.7%)	84 (18.4%)	0.91
Stroke	48 (6.8%)	17 (6.9%)	31 (6.8%)	1.00
Prior MI	77 (10.9%)	27 (10.9%)	50 (11.0%)	1.00
Prior CABG	29 (4.1%)	9 (3.6%)	20 (4.4%)	0.69
LBBB	92 (13.1%)	32 (12.9%)	60 (13.2%)	1.00
Active smoker	21 (3.0%)	6 (2.4%)	15 (3.3%)	0.64
Dyslipidemia	347 (49.3%)	128 (51.6%)	219 (48.0%)	0.38
COPD	54 (7.7%)	20 (8.1%)	34 (7.5%)	0.76
CAD	117 (16.6%)	35 (14.1%)	82 (18.0%)	0.20
DCM	44 (6.2%)	7 (2.8%)	37 (8.1%)	0.005
Creatinine (mg/dl)	1.06 (0.87–1.28)	1.04 (0.84–1.24)	1.07 (0.88–1.29)	0.10
Hemoglobin (g/dL)	12.77 ± 1.77	12.55 ± 1.79	12.89 ± 1.75	0.01
Leucocytes (×10^3^/µL)	6.93 (5.84–8.32)	6.83 (5.81–8.16)	6.99 (5.91–8.37)	0.22
Platelets (×10^3^/µL)	204 (166–244)	203 (163–239.50)	204 (167–247)	0.15
LVEF (%)	50 (41.50–55)	55 (45–55)	50 (40–55)	0.0003
LV diameter (mm)	51 (45–56)	49.78 ± 7.64	51 (46–57)	0.04
Maximum gradient (mmHg)	79 (65–97)	84 (70.50–105.50)	75 (63–90.50)	0.0008
Mean gradient (mmHg)	49 (40–62)	52.50 (43–67)	45 (39.90–57)	0.0001
AVA (cm^2^)	0.67 ± 0.16	0.60 (0.54–0.70)	0.75 ± 0.16	0.009
Annulus area (mm)	463.8 (410.5–532.8)	417.0 (365.0–463.0)	477.2 (420.2–546.5)	0.0001
Annulus perimeter (mm)	77.8 (72.95–83.35)	73.8 ± 6.8	79.1 (74.0–84.3)	0.0001
Minimum annulus diameter (mm)	21.6 (20.0–23.3)	20.0(18.4–21.4)	21.80 (20.4–23.4)	0.0001
Maximum annulus diameter (mm)	27.7 (25.8–29.7)	26.0 ± 2.4	28.1 ± 2.7	0.0001
Sinus of Valsalva diameter (mm)	30.3 (27.9–33.0)	27.7 (26.2–30.2)	30.9 ± 3.3	0.0001
Sinotubular junction diameter (mm)	28.6 (25.8–31)	26.6 (24.8–30.0)	28.9 (26.1–31.0)	0.0016
LCA height (mm)	14.0(12.0–16.5)	12.8 ± 3.2	14.5 (12.4–16.7)	0.0001
RCA height (mm)	17.9 (15.5–20.0)	16.5 (15.0–18.1)	18.0 (16.0–20.0)	0.0001
Sinotubular junction height (mm)	23.5 (21.5–25.6)	22.0 ± 2.9	24.0 (22.0–26.0)	0.0001
EuroSCORE I (%)	8.52 (5.69–14.49)	8.75 (5.43–14.46)	8.35 (5.72–14.49)	0.58
EuroSCORE II (%)	3.83 (2.41–7.70)	3.74 (2.21–6.39)	4.12 (2.74–8.42)	0.14
Maximum transprosthetic gradient (mmHg)	19 (13–27)	19 (14–26)	20 (13–28)	0.64
Mean transprosthetic gradient (mmHg)	10 (7–15)	10 (7–13)	11 (8–15)	0.21

AVA—aortic valve area; CABG—coronary artery bypass graft; CAD—coronary artery disease; COPD—chronic obstructive pulmonary disease; DCM—dilated cardiomyopathy; EuroSCORE—European System for Cardiac Operative Risk Evaluation; HBP—high blood pressure; LBBB—left bundle branch block; LCA—left coronary artery; LV—left ventricle; LVEF—LV ejection fraction; MI—myocardial infarction; RCA—right coronary artery.

**Table 2:** Impact of valve type on survival and pacemaker implantation.

Variable	Number	Percentage (%)	HR	CI Lower 95%	CI Upper 95%	*p*-Value *
All-cause survival prediction						
Pre-dilatation	515	73.1	0.71	0.48	1.04	0.08
Post-dilatation	190	26.9	1.51	1.05	2.19	0.03
ES3	457	64.7	0.67	0.46	0.96	0.03
Accurate	81	11.5	1.07	0.67	1.7	0.77
Boston	59	8.5	0.91	0.28	2.91	0.87
Portico	43	6.1	1.58	0.96	2.6	0.07
Evolut	38	5.4	1.29	0.73	2.26	0.38
Navitor	26	3.6	2.03	0.64	6.48	0.23
Cardiovascular-cause survival prediction						
Pre-dilatation	515	73.1	0.72	0.46	1.14	0.16
Post-dilatation	190	26.9	1.63	1.07	2.50	0.02
ES3	457	64.7	0.66	0.43	1.02	0.06
Accurate	81	11.5	1.04	0.60	1.80	0.88
Boston	59	8.5	0.74	0.18	3.08	0.68
Portico	43	6.1	1.66	0.92	2.96	0.08
Evolut	38	5.4	1.24	0.64	2.42	0.51
Navitor	26	3.6	2.54	0.79	8.19	0.11
Permanent pacemaker implantation						
Pre-dilatation	38	7.69	0.79	0.43	1.43	0.43
Post-dilatation	18	9.94	1.34	0.74	2.42	0.33
ES3	31	7.16	0.69	0.39	1.20	0.19
Accurate	5	6.41	0.74	0.28	1.92	0.54
Boston	5	8.77	1.08	0.41	2.84	0.86
Portico	7	16.2	2.35	1.00	5.57	0.05
Evolut	2	5.41	0.62	0.14	2.68	0.52
Navitor	5	20.8	3.14	1.12	8.75	0.02

CI—confidence interval; ES3—Edwards Sapien3 valve; HR—hazard ratio; OR—odds ratio; * *p*-value was obtained using univariate Cox regression.

**Table 3:** Clinical and anatomic parameters with significant impact on valve selection and pre- and post-dilatation decision in regard to a stepwise multivariable binary logistic regression analysis.

Predictors of Balloon-Expandable Versus Self-Expandable Valve			
Variable	OR	95% CI	*p*-value *
LVEF (%)	0.85	0.75–0.99	0.03
Aortic valve area (cm^2^)	0.97	0.96–0.99	0.01
Aortic regurgitation	4.08	2.04–6.05	0.02
Annulus area (mm^2^)	0.71	0.59–0.86	0.0006
Sinotubular junction diameter (mm)	1.01	1.00–1.01	0.0002
LCA height (mm)	0.89	0.83–0.97	0.004
Predictors of pre-dilatation			
Variable	OR	95% CI	*p*-value *
Dilated cardiomyopathy	0.35	0.18–0.67	0.001
Maximum aortic velocity (m/s)	1.01	1.00–1.02	0.003
Predictors of post-dilatation			
Variable	OR	95% CI	*p*-value *
Coronary artery bypass graft	2.53	1.17–5.48	0.01
Mean aortic gradient (mmHg)	1.01	1.00–1.02	0.004
Aortic regurgitation	1.20	1.00–1.44	0.05
Sinotubular junction height (mm)	0.91	0.85–0.98	0.01

CI—confidence interval; LCA—left coronary artery; LVEF—left ventricular ejection fraction; OR—odds ratio; * *p*-value was obtained using multivariate logistic regression.

## Data Availability

The original contributions presented in the study are included in the article, further inquiries can be directed to the corresponding author.
